# Histopathological Characteristics of Percutaneous Endomyocardial Biopsy in Heart Transplant Rejection Surveillance: A Single Center Experience

**DOI:** 10.3390/biomedicines12102258

**Published:** 2024-10-04

**Authors:** Anca Otilia Farcas, Mihai Ciprian Stoica, Septimiu Voidazan, Ioana Maria Maier, Adrian Cornel Maier, Horatiu Suciu, Anca Ileana Sin

**Affiliations:** 1 Doctoral School of Medicine and Pharmacy, George Emil Palade University of Medicine, Pharmacy, Sciences and Technology of Targu Mures, 540142 Targu Mures, Romania; ancacontac@gmail.com; 2 Department of Cell Biology, George Emil Palade University of Medicine, Pharmacy, Science and Technology of Targu Mures, 540139 Targu Mures, Romania; ileana.sin@umfst.ro; 3 Department of Nephrology/Internal Medicine, Mures County Clinical Hospital, 540103 Targu Mures, Romania; 4 Department of Internal Medicine, George Emil Palade University of Medicine, Pharmacy, Sciences and Technology of Targu Mures, 540139 Targu Mures, Romania; 5 Epidemiology Department, University of Medicine, Pharmacy, Science and Technology ‘George Emil Palade’ of Târgu Mureş, 540139 Targu Mures, Romania; 6 Emergency Military Hospital, 800150 Galati, Romania; mioanamaier@gmail.com; 7 Faculty of Medicine and Pharmacy, Dunarea de Jos University, 800008 Galati, Romania; 8 Department of Surgery M3, George Emil Palade University of Medicine, Pharmacy, Science and Technology of Targu Mures, 540139 Targu Mures, Romania; 9 Emergency Institute for Cardiovascular Diseases and Transplantation Targu Mures, 540136 Targu Mures, Romania; 10 Department of Pathology, Clinical County Emergency Hospital, 540136 Targu Mures, Romania

**Keywords:** heart transplant rejection, endomyocardial biopsy (EMB), histopathology, acute cellular rejection (ACR), antibody mediated rejection (AMR), Quilty effect Q.E., fibrosis, vasculitis

## Abstract

Background: Heart transplantation (HT) remains the ultimate treatment for end-stage heart failure. An endomyocardial biopsy (EMB) is “the gold standard” diagnostic procedure used in HT rejection surveillance. The aim of this study is to provide a detailed analysis of the histopathological characteristics of the EMB and to investigate if there is a correlation between some histopathological changes, such as fibrosis, vasculitis, Quilty effect (Q.E.), myocytes damage, and the presence of episodes of acute rejection. Methods: In this retrospective study, 200 EMBs were included, coming from 65 patients transplanted in the Emergency Institute for Cardiovascular Diseases and Transplantation (ICvDT) Targu Mures between 2012 and 2024. Fibrosis, vasculitis, Q.E., myocyte damage, etc., were microscopically evaluated to see if these parameters correlate with rejection episodes. Results: The mean age was 38.18 years (SD 15.67), 25% of biopsies being recorded in the 41–50 age group. 77.14% of total acute cellular rejection (ACR) was of mild rejection, with most registered in the 11–20 age group; the cases of severe rejection being recorded in the 41–50 age group. Antibody-mediated rejection (AMR) was recorded more frequently in women with a representation of 23.4%, compared to 8.5% of men. 86.7% (39 cases) of the total number of EMBs with fibrosis score 3 and 71.4% (15 cases) of the total EMBs with fibrosis score 2 were recorded in men, compared to the 28.6% (6 cases) of fibrosis score 2 recorded in women (*p* = 0.013). 50.0% of all the EMB recorded in the 61–70 age group showed fibrosis score 3, compared to 34.8% of those from the 21–30 age group. The Q.E. was identified in 13% of the biopsies and, in some patients, it was observed across 3–4 successive biopsies. Mild vasculitis was associated in 34.9% of cases with ISHLT ≥ 1R and moderate vasculitis was associated in 87.5% of cases with ISHLT ≥ 1R. Conclusions: Fibrosis was detected much more frequently in men and in the 61–70 age group. In addition to the histopathological changes specific to acute rejection, there are other pathological changes, such as the Q.E., and vasculitis and myocytes damage and disarray, that seem to suggest a close connection with rejection, but extensive studies are needed to confirm this.

## 1. Introduction

Heart transplantation remains the ultimate treatment for end-stage heart failure (functional class NYHA III-IV, despite maximal therapy), [1] often providing an increased quality of life compared to the pre-transplantation quality of life. However, the numerous complications associated with transplantation or caused by immunosuppressive treatment, such as infections, malignancies, renal failure, or rejection episodes, reduce the survival rate and the quality of life of transplanted patients.

A percutaneous EMB is a critical diagnostic procedure in the surveillance of heart transplant rejection. As heart transplantation becomes an increasingly viable treatment for end-stage heart failure [2], the long-term success of these procedures relies heavily on the early detection and management of allograft rejection. A common post-transplant complication is represented by rejection that can lead to graft dysfunction and, if undetected, graft failure. An EMB involves the sampling of myocardial tissue from the transplanted heart, typically performed through a right internal jugular or femoral venous approach [3], or in case of its thrombosis, the left internal jugular approach being an alternative [4]. This procedure requires fluoroscopic or echocardiographic guidance [5,6]. The biopsied tissue is then analyzed histologically to identify possible signs of rejection, such as cellular infiltration, myocyte damage, or vascular and interstitial changes. Despite the emergence of non-invasive methods, an EMB remains the gold standard for diagnosing acute rejection, providing direct and specific information about the histopathological state of the myocardium. This technique not only aids in the timely diagnosis of rejection but also guides immunosuppressive therapy, ensuring optimal outcomes for transplant recipients. 

Over time, there have been numerous systems for histopathological classification and grading for heart transplant rejection. Still, the current system used for grading and quantifying rejection is the one proposed by the International Society for Heart and Lung Transplantation (ISHLT) in 2004 for cellular rejection [7] and the one proposed in 2013 for antibody-mediated rejection [8]. 

Although there are many non-invasive diagnostic methods [9,10,11,12] that have proven their usefulness in the diagnosis of acute cellular or humoral rejection, the association of these non-invasive methods with endomyocardial biopsies remains, at least for a while, the “gold standard” in the monitoring of HT patients [13]. 

In addition to the specific changes related to rejection (inflammatory response of the host against the transplanted heart), many other changes not related to rejection can be found in an EMB; thus, detailed knowledge of these aspects and their correct interpretation is vital in order to avoid misdiagnosing them as rejection.

In Târgu Mureș, the first HT was performed in 1999—98 patients benefiting from heart transplantation in this center—during the period 1999–2024. The first EMB was performed also in 1999. Because rejection symptoms may appear late due to the denervation of the transplanted heart [14], constant monitoring of rejection is necessary. In our center, an EMB is performed one month, six months, and one year post-transplant, then once a year in the first 5 years post-transplant. An EMB is also performed whenever there is a clinical suspicion of rejection. 

The purpose of this study is to provide a detailed analysis of the histopathological characteristics of endomyocardial biopsies performed in heart transplant patients to identify rejection as well as highlight the histopathological changes that occur in the myocardial tissue that are not associated with rejection and to investigate if there is a correlation between some histopathological changes such as fibrosis, vasculitis, Quilty effect (Q:E), and myocytes damage, and the presence of episodes of acute rejection.

## 2. Materials and Methods

In this retrospective single-center study, we evaluated an EMB and the histopathological reports of HT patients from the ICvDT that occurred in Mures County between 2012 and 2024. Sixty-five (65) HT patients were included in the study, whose transplant was performed in ICvDT Targu Mures, Romania thus obtaining 200 EMBs for monitoring rejection in these patients. Since both pediatric and adult transplants are performed in this transplant center, both age categories were included in the study. We excluded from the study the patients whose transplantation was performed in this center, but whose surveillance EMBs were performed in another center, as well as the patients whose death occurred immediately post-operatively, without performing any EMBs. The study was approved by The Ethics and Research Committee of Clinical County Emergency Hospital, Targu Mures, Romania, under the Declaration of Helsinki.

In this transplant center, the immunosuppressive treatment was administered according to the recommendations of the ISHLT protocols [13] and the induction treatment was administered preoperatively to all patients.

For the surveillance of HT patients, the standard protocol for performing EMBs was used: in a cardiac catheterization room, under fluoroscopic guidance and monitoring heart rate, with oxygen saturation and non-invasive blood pressure, under local anesthesia with lidocaine, a bioptome is introduced to the internal jugular vein and is advanced through a sheath that previously had been placed using the standard Seldinger technique. 

In the microscopical evaluation of EMBs, the following parameters were evaluated to identify possible correlations between them and rejection episodes: vasculitis, myocyte damage, the appearance of small intramyocardial vessels, interstitial and perivascular fibrosis, inflammatory infiltrate, and the Q.E. Other microscopic aspects evaluated in this study were histopathological changes suggestive of CMV infection, vasculopathy and other lesions not related to rejection (hemorrhage, presence of adipocytes).

ACR is defined as the presence of focal or diffuse interstitial or perivascular lymphocytic infiltrate (T-cells) leading to myocyte damage, and the alteration of the normal architecture of cardiac muscle fibers and necrosis, even producing cardiac allograft dysfunction in severe forms of rejection. According to the ISHLT 2004 classification, it is divided into 4 grades: ISHLT 0, corresponding to the absence of ACR; ISHLT 1R (mild rejection), characterized by interstitial and/or perivascular infiltrate with up to 1 focus of myocyte damage; ISHLT 2R (moderate ACR), characterized histopathologically by two or more foci of predominant lymphocytic infiltrate with associated myocyte damage; and ISHLT 3R (severe ACR) when diffuse predominant lymphocitic infiltrate is associated with multifocal myocyte damage, ±edema, ±hemorrhage, or ±vasculitis [7].

AMR is defined as rejection mediated by B lymphocytes, which secrete antibodies against antigens such as the proteins on the surface of cardiac allograft cells. According to the ISHLT classification from 2013, AMR is divided into 5 grades as follows: pAMR 0—absence of humoral rejection; pAMR 1 (H+), characterized by the presence of histological AMR, but absent immunohistochemically; pAMR 1 (I+), characterized by the absence of histological changes of AMR, but confirmed by positive immunohistochemistry; pAMR 2 when there are both histological changes characteristic of AMR and positive immunohistochemical reactions; and pAMR 3, characterized histopathologically by interstitial hemorrhage, capillary fragmentation, mixed inflammatory infiltrates, endothelial cell pyknosis, and/or karyorrhexis and marked edema [8].

### Statistical Analysis

For the statistical analysis of the data included in this study, we worked with SPSS software version 23. In the statistical analysis, qualitative data was presented as counts and percentages. The association between qualitative variables was assessed using the Chi-square test or the Fisher exact test. The normal distribution of data was checked with a strip-chart, quantile–quantile plot, and Shapiro–Wilk test. Quantitative data was presented as the mean and standard deviation (for normally distributed data) or by median and minimum–maximum or interquartile range (for non-normally distributed data). For all the statistical tests, the significance level alpha was set at 0.05 and the two-tailed *p* value was computed.

## 3. Results

Sixty-five (65) transplanted patients (eight transplants registered in children and 57 in adults) were included in this study, 200 EMBs were performed on them in order to monitor allograft rejection. The mean age of the patients included in the study was 38.18 years (SD 15.67), for the pediatric patients the mean age was 14.75 (SD 2.29) years, and for the adult patients the mean age was 43.8 (SD 11.69) years. The youngest registered age was 11 years old and the oldest age was 67; 25% of the total biopsies were recorded in the age group of 41–50 years. Other studies such as those of Vicenzo Cusi et al. [15] or Craig R. Butler et al. [16] report higher mean ages of 55.5 years and 51 years, respectively. Among the 65 patients, 76.92% are represented by men, with an average number of biopsies/patient of approximately three biopsies (a minimum of one and a maximum of 10 biopsies). The mean number of harvested/collected fragments/biopsies was 3.92 (a minimum of one and a maximum of seven).

The mean follow-up was 58.95 months (SD 30.6 months), with mortality throughout the study being comparable for both sexes: 20% among women (three deaths) 22% among men (11 deaths).

**Figure 1:**
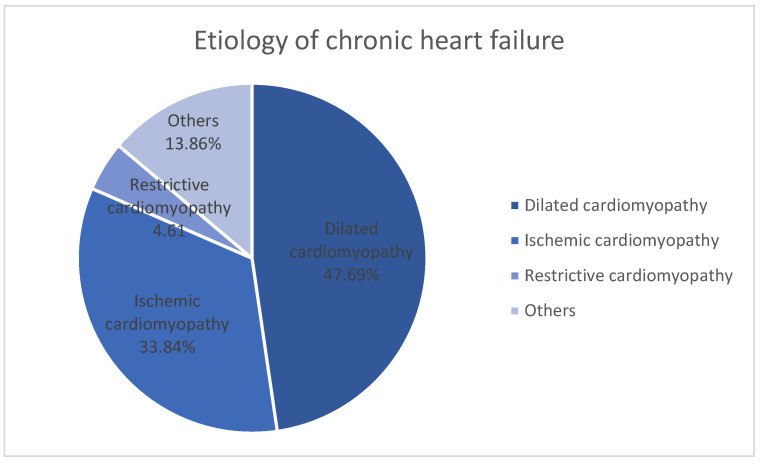
Etiology of chronic heart failure.

The etiology of chronic heart failure was severe dilated cardiomyopathy in 47.69% (31 cases) of the patients, followed by ischemic cardiomyopathy in 33.84% of the patients (22 cases), restrictive cardiomyopathy in 4.61% of the patients (3 cases), and other causes in 13.84% of the patients (nine cases) (Figure 1).

In order to be able to identify histopathological changes present in certain age groups, the patients included in this study were divided into six age groups as follows: group 1: between 11 and 20 years (44 cases); group 2: between 21 and 30 years (23 cases); group 3: 31–40 years (32 cases); group 4: 41–50 years (50 cases); group 5: 51–60 years (37 cases) and group 6: 61–70 years (14 cases). 

Among the 35 cases of acute cellular rejection, 27 cases (77.14%) were of mild rejection, which predominantly affected the age group between 11 and 20 years—eight cases—(29.6%) with the cases of severe rejection being recorded entirely in the age group of 41–50 years (two cases). The most affected age group by episodes of moderate rejection was the age group of 11–20 years; 66.7% of the total episodes were recorded in this age group. 

Regarding the gender distribution of episodes of acute cellular rejection (ACR), 23.40% of all the EMBs taken from women and 15.68% of biopsies taken from men present ACR. Women and men were similarly affected by mild rejection episodes. For example, 14.9% of the cases (seven cases) occurred in women, compared to 13.1% (20 cases) in men, while episodes of moderate rejection occurred more frequently in women at 6.4% (three cases), compared to 2.0% in men (3 cases); episodes of severe rejection affected 2.1% of women and 0.7% of men, but this difference was not considered statistically significant (*p* = 0.293).

Episodes of antibody-mediated rejection (AMR) were recorded more frequently in women, comprising 23.4% of the episodes (11 cases), compared to 8.5% (13 cases) of the episodes recorded in men, with a significant *p* value (*p* = 0.01). The most affected age group was the 11–20-year-old age group with a percentage of 29.5% (13 cases), followed by the 21–30-year-old age group with 21.7% (five cases). At the opposite pole is the 61–70-year-old age group, without any recorded episodes of AMR (*p* = 0.01).

Of the 26 EMBs that showed the Q.E., 11 were recorded among women, with 23.4% of the total biopsies taken from them showing this change as unrelated to rejection, compared to 9.8% of the biopsies taken from men (*p* = 0.024). An interesting aspect observed in this study is the fact that of the 26 cases of the Q.E., 12 (46.2% of the total) were registered in the age group of 11–20 years, but this histopathological aspect was noy registered in the age group of 61–70 years (*p* = 0.026). 

**Figure 2:**
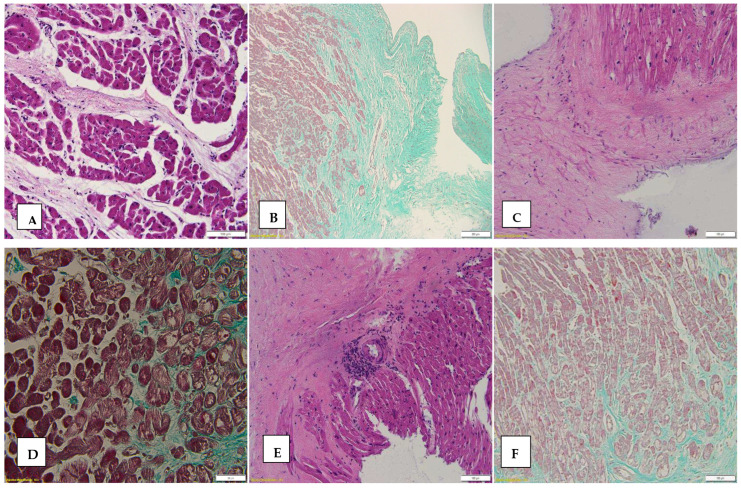
Interstitial fibrosis on EMB. (**A**) Bundles of collagen on HE stain, ob 20×); (**B**) interstitial fibrosis on Masson trichrome stain (ob 10×). (**C**) Marked fibrosis leading to the disorganization of the normal architecture of cardiac tissue (HE stain ob 20×). (**D**) Interstitial fibrosis, Masson trichrome stain, ob 40×. (**E**) Marked fibrosis with the production of architectural disorganization in cardiac tissue HE, ob 4×. (**F**) Fine frames of interstitial fibrosis can be visualized predominantly in the left half of the image, Masson trichrome.

To quantify the microscopic appearance of interstitial fibrosis, we performed special stainings to highlight collagen fibers, such as Masson trichrome staining and Van Gieson staining, using the following scale: score 0—absence of fibrosis; score 1 for the presence of fine fibrosis frames (collagen fibers with a thickness of up to 15 microns) and focal fibrosis; score 2 for the presence of thicker collagen frames (collagen fibers with a thickness between 16 and 30 microns), with a compact distribution within the myocardium, and score 3 for the presence of collagen bands (collagen bands with a thickness greater than 30 microns), with a diffuse distribution. A statistically significant difference in the severity of fibrosis was found for score 3 (the presence of bands of fibrosis); these were present in men in a percentage of 86.7% (39 cases) of the total number of cases of fibrosis score 3. Also, “moderate” fibrosis (score 2) was present more frequently in men by 71.4% (15 cases) compared to the 28.6% (six cases) recorded in women (*p* = 0.013). Figure 2 shows different aspect of interstitial fibrosis.

Regarding the distribution of fibrosis by age group, “severe”, diffuse fibrosis (score 3) was most frequently found in the 61–70 age group, with 50.0% of all the EMBs recorded in this age group showing fibrosis score 3 compared to 37.5% (12 cases) of those taken from the age group of 31–40 years and 34.8% (7 cases) of those taken from the age group of 21–30 years. In the age group of 11–20 years, the presence of fibrosis score 3 was found at a percentage of only 13.6% of the total biopsies taken from this age group (*p* = 0.047).

Another histopathological aspect evaluated on the EMBs was vasculitis, which was graded as mild vasculitis and moderate vasculitis. This was found in 36.17% (17 cases) of the total biopsies taken from women, 12.8% being moderate vasculitis, compared to 22.22% (34 cases) of the total biopsies taken from men. In the latter, moderate vasculitis was present in only 1.3% of the biopsies, with a statistically significant *p* value (*p* = 0.003).

Table 1 shows in detail the gender distribution of the parameters discussed above (ACR, AMR, Q.E., fibrosis, vasculitis).

For the quantitative evaluation of the inflammatory infiltrate present on the EMB, scores from zero to three were used as follows: 0 = absence of inflammatory infiltrate; score 1 = minimal mononuclear inflammatory infiltrate; score 2 = inflammatory infiltrate in a moderate amount; score 3 = abundant, diffuse inflammatory infiltrate. A minimal amount of mononuclear inflammatory infiltrate (score 1) was found on the majority of the EMBs taken from women (57.4%), as well as those taken from men (69.3%); this difference was not statistically significant (*p* = 0.42), but the absence of the diffuse, abundant inflammatory infiltrate was noted in the extreme age groups 11–20 years and 61–70 years, as well as in the 51–60 age group.

Among the histopathological aspects that can be observed in the case of myocyte damage, in our study, we evaluated the following: the appearance of the cytoplasm (premiocytolysis, microvacuolations, hypereosinophilia, finely granular cytoplasm, hydropic dystrophy), the architectural aspect (hypertrophy of cardiomyocytes, coagulation necrosis, myocyte disaggregation), and the integrity of the nucleus. Thus, the normal appearance of the cardiomyocytes was quantified with score zero, the presence of a single change among those listed above was quantified with score 1, the presence of two or three associated changes was quantified with score 2 and score 3, respectively, for the association of more than three microscopic aspects of those listed above. 

A percentage of 10.6% of biopsies taken from women and 7.8% of those taken from men did not show microscopic changes detectable in the usual hematoxylin–eosin staining at the level of cardiomyocytes. Relatively similar percentages were obtained in the case of the myocytic damage score 2 (51.1% of the biopsies taken from women, 54.9% of those taken from men). Significant differences were recorded for the myocytic damage score 1, with men being almost 4 times more affected than women (24.2% among the biopsies taken from men compared to 6.4% among the biopsies from women). At the opposite pole, the myocytic damage score 3 was much more frequently found among women (31.9%) compared to men (13.1%) (*p* = 0.004).

Regarding the myocytic damage related to the age groups, it was found that the entirety of the 61–70-year-old age group was affected by pathological microscopic changes present in the cardiac tissue; in this age group, no EMBs with a normal histological aspect was recorded (14/14 EMB shows at least the myocytic damage score 1). However, the age group with the most frequent score 3 impairment was the 11–20-year-old age group (36.4%), followed by the age group of 21–30 years (26.1%) (*p* = 0.003).

Another parameter evaluated in this study was the appearance of small intramyocardial vessels, with pathological changes recorded, such as swelling of the endothelium, thickening of the intima, dystrophic endothelial lesions, intramural edema, perivascular edema, the presence of foamy histiocytes, and the presence of intramural and perivascular inflammatory infiltrate. A stratification similar to that performed for “myocyte damage” was carried out. Regarding the distribution by gender and age group, no statistically significant differences were observed (*p* = 0.25, 0.76); however, a close association was found between the ISHLT grade and the score for the histopathological changes at the level of small intramyocardial vessels. Both sexes and age groups were relatively uniformly affected by these changes in small intramyocardial vessels (*p* = 0.001). Thus, score 3 was observed in 100% of ISHLT 3R cases, but only 9.1% of ISHLT 0 biopsies were associated with small vessel changes quantified with score 3.

Microscopic changes suggestive of CMV infection manifested by the presence of cells with large nuclei and lymphoplasmacytic inflammatory infiltrate around or by the presence of cells with enlarged nuclei, with rough chromatin, were recorded in 2.1% (one case) of the endomyocardial biopsies taken from women and 3.9% of those from men (six cases). Of the seven registered cases, two were present in the 11–20 age group, two in the 41–50 age group, two in the 51–60 age group, and one case in the 61–70 age group.

## 4. Discussion

In this retrospective single-center study, between January 2012 and March 2024, 200 routine surveillance endomyocardial biopsies were performed and analyzed, these coming from 65 transplanted patients, reducing as much as possible the number of “unnecessary” EMBs, with an average number of biopsies/patient of approximately three biopsies/patient. Compared to the results from our study, other authors have reported a higher average number of biopsies/patient, between 5.9 and more than 10 biopsies/patient [17,18,19]. 

The patients were followed between a minimum of one month and a maximum of 144 months, the average being approximately 59 months; to date/at the time of writing, the survival rate among women is 80% and 78% among men. In their study, Ghadimi H et al. [20] reported a survival of 80% at one month and 50% at 5 years. Trivedi, Jaimin R et al. [21], in their study in which 17,131 patients were included and evaluated based on their survival at 1 and 5 years, based on a recipient-donor risk score, the reported survival rates at 5 years were between 81% and 64%, depending on the recipient’s group and risk, which is data similar to those of our study.

The most frequent etiology of end-stage heart failure for which transplantation is resorted to, mentioned in numerous studies, is ischemic cardiomyopathy with rates varying between 58% [2] and 32.4% [22,23]. In our study, heart failure caused by ischemic heart disease was the second most common etiology 33.84%, preceded by dilated cardiomyopathies (47.69%). 

Histopathologically, ACR transposes through the presence of predominantly lymphocytic inflammatory infiltrate that often leads to myocyte injury [7]. In our study, the rate of mild rejection episodes (ISHLT 1R) was 13.5%, that of moderate rejection episodes (ISHLT 2R) was 3%, and severe rejection episodes (ISHLT 3R) represented 1% of the total number of biopsies. Other studies reported a rate of ISHLT 1R between 22.8% and 34% [2,24,25]. In 13 of the 27 cases ISHLT 1R (48.1%), ACR was associated with pAMR, all six ISHLT 2R biopsies and two ISHLT 3R biopsies were associated with pAMR.

The Q.E. is nodular lymphocytic infiltrates localized within the endocardium. Histopathologically, it is predominantly made up of B lymphocytes, admixed with plasma cells, macrophages, and dendritic cells, among which small capillaries can be observed, giving the appearance of a lymphoid organ [26]. These are generally considered benign, but there are numerous studies that suggest a close connection between rejection episodes and the Q.E. [27]. However, their clinical significance is debated, particularly when they are persistent [26]. While Quilty lesions are not directly associated with graft rejection, their presence can complicate the histopathological interpretation of EMBs. This is because Quilty lesions can mimic low-grade rejection, potentially leading to over-treatment if not accurately identified. The recurrence of the Q.E. in successive biopsies raises questions about its pathogenesis and whether it represents a sustained immune response [28] or an artifact of the healing process. 

In our study, the Q.E. was identified in 13% of the biopsies (26 biopsies), corresponding to 12 patients. Interestingly, in some patients, these lesions were observed across 3–4 successive biopsies. Among the 12 patients in whom the Q.E. was observed, four were pediatric patients, two of them having four successive affected biopsies each, with the other two pediatric patients having three successive affected endomyocardial biopsies each. Unfortunately, one of the pediatric patients with four successive biopsies in which the Q.E. was observed also presented repeated episodes of pAMR(H+), and the patient succumbed to their illness.

**Figure 3:**
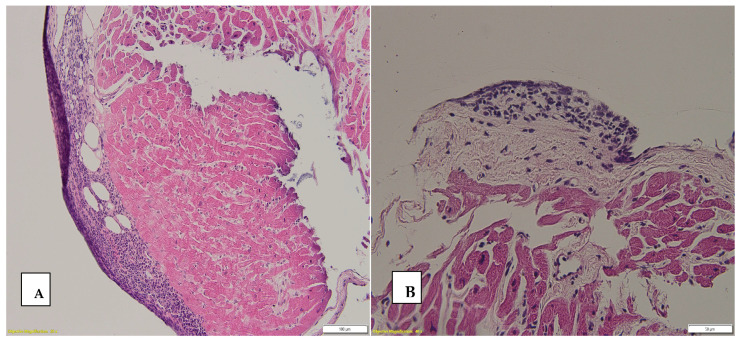
Quilty effect ((**A**) Q.E. and subendocardial fat tissue, HE stain ob 10× (**B**) Q.E., HE stain ob 20×).

Of the 26 biopsies with the Q.E., 38.5% (10 biopsies) also showed ACR ISHLT 1R and 7.7% (two biopsies) showed ACR ISHLT 2R (*p* = 0.001). In the case of humoral rejection, the Q.E. was associated almost four times more frequently with pAMR (37.5%) compared to biopsies in which the presence of the Quilty effect was found, but without humoral rejection (9.7%) (*p* = 0.001). Figure 3 shows the Q.E.

Interstitial and scarring fibrosis were common findings in our biopsies, being present in 76% (152) of the biopsies, with 63% (126 biopsies) having no signs of acute cellular or humoral rejection (ISHLT 0). In their study, Kae Watanabe et al. [29] found that interstitial fibrosis associated with microvessel disease in pediatric patients with CAV, was present much more frequently compared to the presence of these changes in the control group (pediatric heart transplant patients without CAV). An interesting fact is that these changes were visible in many cases on EMBs, before CAV was diagnosed. Also, in our study, it was found that the more frequent association of vascular lesions quantified with score 2 and score 3 with ACR: 60.5% of biopsies with vascular changes of score 3 had associated ISHLT ≥ 1R, while 46.3% of biopsies with score 2 associated with ISHLT ≥ 1R.

Fibrosis can result from repeated episodes of rejection, ischemic injury [30], or other forms of graft damage, a possible physiopathological mechanism being the direct activation of fibroblasts by a subset of T lymphocytes through the production of profibrotic cytokines, interleukin 4, and interleukin 13 [30]. Its presence is indicative of ongoing or past myocardial injuries and can contribute to long-term graft dysfunction. In light of what has been presented, fibrosis should not be seen as a benign process, but, rather, as a signal of chronic injury to the graft.

**Figure 4:**
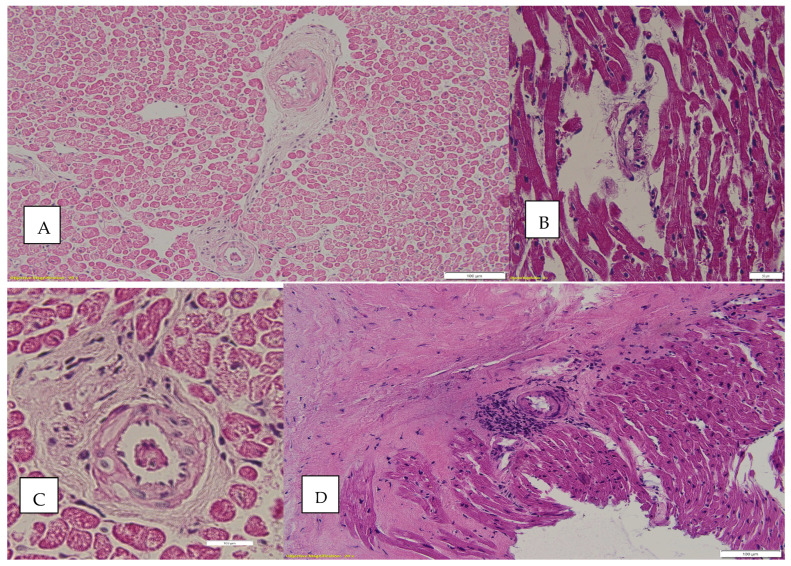
(**A**) Vascular changes, HE Ob 20×; (**B**) mild Vasculitis, HE Ob 20×; (**C**) vascular changes, HE, Ob 40×; (**D**) perivascular inflammatory infiltrate and thickening of the media.

There are numerous studies, including the one led by Hui Tzu Lin-Wang [31], in which the association of rejection episodes with vasculitis was confirmed, but there are entities such as post-transplant lymphoproliferative disorder (PTLD) that can mimic vasculitis when coronary or intracardiac vessels are affected [32]. Therefore, it is necessary to examine the inflammatory infiltrate with particular attention when it is present in endomyocardial biopsies. In our study, mild vasculitis was associated with 34.9% of the cases with ISHLT ≥ 1R and moderate vasculitis was associated with 87.5% of the cases with ISHLT ≥1R; 91.3% of the biopsies in which the absence of vasculitis was found were ISHLT 0. Figure 4 shows some of the pathological changes found in the intracardiac vessels.

**Figure 5:**
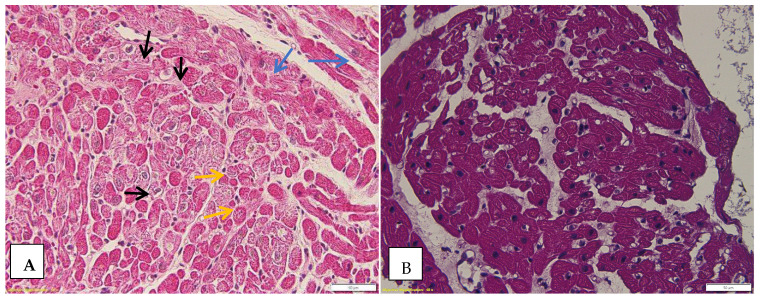
Cardiomyocytes changes. (**A**) Black arrow—perinuclear halo; blue arrow—hypertrophic fibers; yellow arrow—fine granular cytoplasm; HE, Ob 40×. (**B**) Cardiomyocytes disarray can be seen as can a perinuclear halo in some cardiac fibers.

In the normal heart tissue, cardiomyocytes disarray is not common [33], a fact supported by the results of our study in which it was found that in 100% of the cases with severe rejection (ISHLT 3R) it was associated with the myocytic damage score 3, 57.1% of ISHLT 2R biopsies were associated with the myocytic damage score 3, and 37.5% of ISHLT biopsies 1R also presented the myocytic damage score 3. At the opposite pole, 94.1% of the ISHLT 0 biopsies did not have associated myocytic damage (score 0). Figure 5 shows some of the microscopic changes found in cardiomyocytes.

Other histopathological changes found on the EMBs were the presence of intraventricular adipose tissue, intravascular microthrombi, interstitial edema, and endocardial thickening, but for these aspects, no associations with rejection episodes were found. There were no cases of hemorrhage, granulocytic inflammatory infiltrate, or PTLD visualized on the EMBs.

## 5. Conclusions

EMBs remain an important diagnostic tool in the diagnosis and monitoring of heart transplant rejection. The EMB procedure is safe, depending on the experience of the operating team, but in our center, there were no complications after this invasive procedure. Fibrosis was detected much more frequently in men and in the 61–70 age group. Women were more frequently affected by the myocytic damage score 3 compared to men, who more frequently presented the myocytic damage score 1. The Q.E was associated almost 4 times more frequently with pAMR compared to biopsies in which the presence of the Q.E. was found, but without humoral rejection. In addition to the histopathological changes specific to acute cellular and humoral rejection, there are other pathological changes, such as the Q.E. and vasculitis, and microscopic changes occurring at the level of cardiomyocytes, that seem to suggest a close connection with rejection episodes, but extensive studies are needed to confirm this.

## Figures and Tables

**Table 1:** Gender and age group distribution of ACR, AMR, Quilty effect, fibrosis, and vasculitis.

Parameter		SEX		*p* Value	Age Group						*p* Value
		F	M		11–20 y.o.	21–30 y.o.	31–40 y.o.	41–50 y.o.	51–60 y.o.	61–70 y.o.	
ISHLT	ISHLT 0	76.6% (36)	84.3% (129)	*p =* 0.293	72.7% (32)	73.9% (17)	84.4% (27)	86.0% (43)	89.2% (33)	92.9% (13)	*p =* 0.192
	ISHLT 1R	14.9% (7)	13.1% (20)		18.2% (8)	21.7% (5)	15.6% (5)	8.0% (4)	10.8% (4)	7.1% (1)	
	ISHLT 2R	6.4% (3)	2.0% (3)		9.1% (4)	4.3% (1)	0.0% (0)	2.0% (1)	0.0% (0)	0.0% (0)	
	ISHLT 3R	2.1% (1)	0.7% (1)		0.0% (0)	0.0% (0)	0.0% (0)	4.0% (2)	0.0% (0)	0.0% (0)	
Antibody Mediated Rejection	Absent	76.6% (36)	91.5% (140)	*p =* 0.010	70.5% (31)	78.3% (18)	96.9% (31)	92.0% (46)	97.3% (36)	100.0% (14)	*p =* 0.01
	Present	23.4% (11)	8.5% (13)		29.5% (13)	21.7% (5)	3.1% (1)	8.0% (4)	2.7% (1)	0.0% (0)	
Quilty Effect	Absent	76.6% (36)	90.2% (138)	*p =* 0.024	72.7% (32)	82.6% (19)	93.8% (30)	92.0% (46)	89.2% (33)	100.0% (14)	*p =* 0.026
	Present	23.4% (11)	9.8% (15)		27.3% (12)	17.4% (4)	6.2% (2)	8.0% (4)	10.8% (4)	0.0% (0)	
Fibrosis	Score 0	34.0% (16)	20.9% (32)	*p =* 0.013	36.4% (16)	21.7% (5)	12.5% (4)	26.0% (13)	21.6% (8)	14.3% (2)	*p =* 0.047
	Score 1	40.4% (19)	43.8% (67)		38.6% (17)	30.4% (7)	37.5% (12)	52.0% (26)	54.1% (20)	28.6% (4)	
	Score 2	12.8% (6)	9.8% (15)		11.4% (5)	13.0% (3)	12.5% (4)	12.0% (6)	5.4% (2)	7.1% (1)	
	Score 3	12.8% (6)	25.5% (39)		13.6% (6)	34.8% (8)	37.5% (12)	10.0% (5)	18.9% (7)	50.0% (7)	
Vasculitis	Absent	63.8% (30)	77.8% (119)	*p =* 0.003	65.9% (29)	73.9% (17)	65.6% (21)	88.0% (44)	73.0% (27)	78.6% (11)	*p =* 0.21
	Mild Vasculitis	23.4% (11)	20.9% (32)		31.8% (14)	17.4% (4)	25.0% (8)	10.0% (5)	24.3% (9)	21.4% (3)	
	Moderate Vasculitis	12.8% (6)	1.3% (2)		2.3% (1)	8.7% (2)	9.4% (3)	2.0% (1)	2.7% (1)	0.0% (0)	

## Data Availability

The data presented in this study are available on request from the corresponding author due to privacy, legal reasons and ethical reasons.
